# Supplementary material for: Identification of Fis1 Interactors in Toxoplasma gondii Reveals a Novel Protein Required for Peripheral Distribution of the Mitochondrion
Source: mBio. 2020 Feb 11;11(1):e02732-19. doi: 10.1128/mBio.02732-19 (PMC7018656; doi:10.1128/mBio.02732-19)
Supplement: FIG S1 [file mBio.02732-19-sf001.pdf]

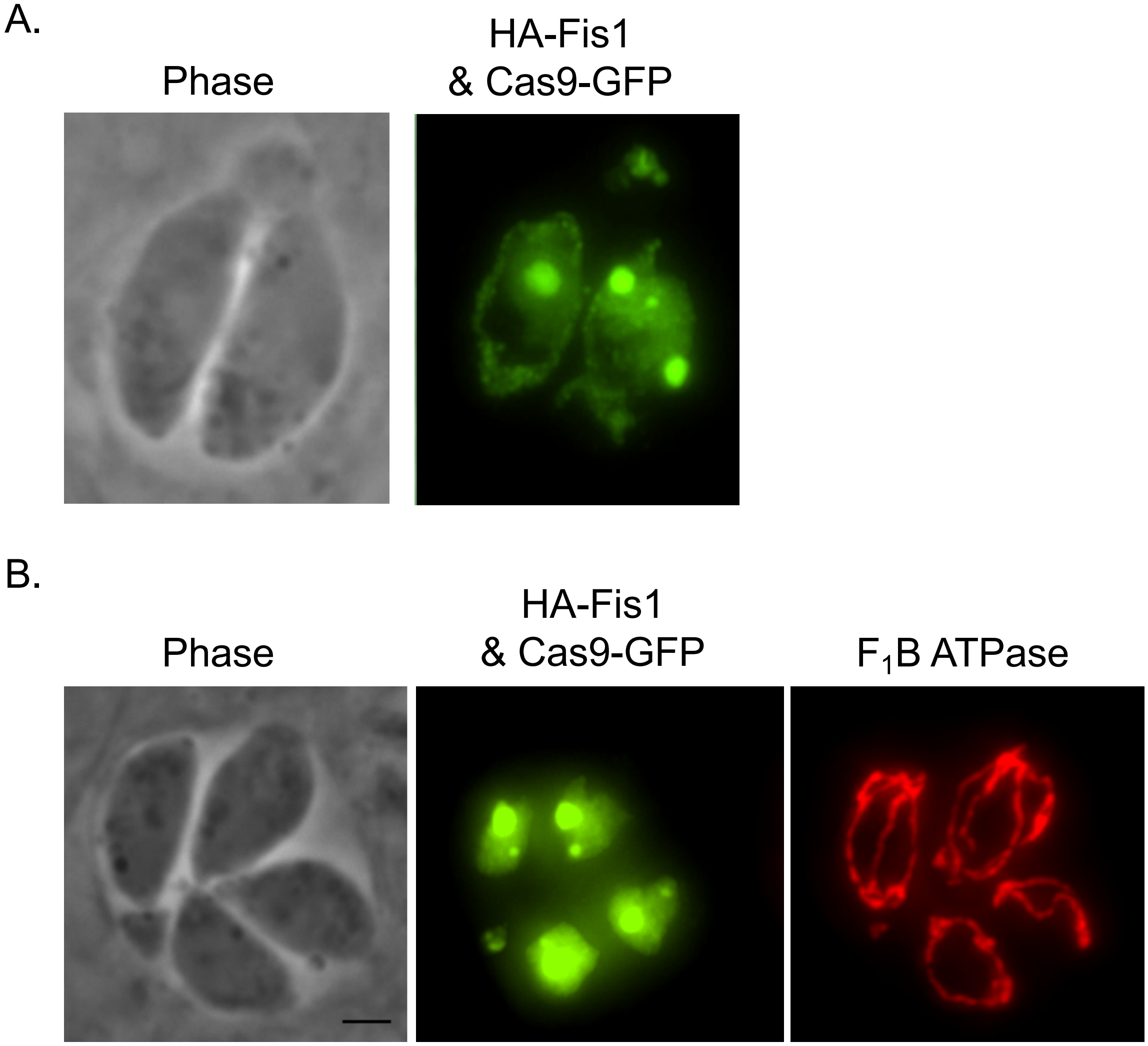

Supplemental Figure S1. Parasites of the HA-Fis1 expressing strain were transfected with a vector encoding Cas9-GFP and a Fis1 specific guide RNA. Parasites were stained for HA to detect Fis1. Nuclear GFP signal indicates incorporation of vector and expression of Cas9. (A) some parasites expressing Cas9 retain Fis1 signal (A) while others do not (B). The morphology of the mitochondrion for parasites not expressing Fis1 was monitored by staining for F_1BATPase.
