# Supplementary material for: Identification of Fis1 Interactors in Toxoplasma gondii Reveals a Novel Protein Required for Peripheral Distribution of the Mitochondrion
Source: mBio. 2020 Feb 11;11(1):e02732-19. doi: 10.1128/mBio.02732-19 (PMC7018656; doi:10.1128/mBio.02732-19)
Supplement: TABLE S1 [file mBio.02732-19-st001.pdf]

Protein ID	Product annotation
TGGT1_241170	hypothetical protein
TGGT1_248520	hypothetical protein
TGGT1_265180	hypothetical protein
TGGT1_305270	hypothetical protein
TGGT1_410610	hypothetical protein
TGGT1_227850	peptidyl-prolyl cis-trans isomerase, cyclophilin-type domain-containing protein
TGGT1_356400	cAMP-dependent protein kinase
TGGT1_305940	peptidyl-prolyl cis-trans isomerase, cyclophilin-type domain-containing protein
TGGT1_211670	S1 RNA binding domain-containing protein
TGGT1_213670	hypothetical protein
TGGT1_212090	hypothetical protein

Table S1. Proteomics results from immunoprecipitation (IP) of HA-Fis1. Shown are proteins that had 5 or more peptides in IP from the HA-Fis1 expressing strain and none in the parental strain. Highlighted is the protein also identified through the yeast two-hybrid screen.
