# Supplementary material for: Identification of Fis1 Interactors in Toxoplasma gondii Reveals a Novel Protein Required for Peripheral Distribution of the Mitochondrion
Source: mBio. 2020 Feb 11;11(1):e02732-19. doi: 10.1128/mBio.02732-19 (PMC7018656; doi:10.1128/mBio.02732-19)
Supplement: FIG S2 [file mBio.02732-19-sf002.pdf]

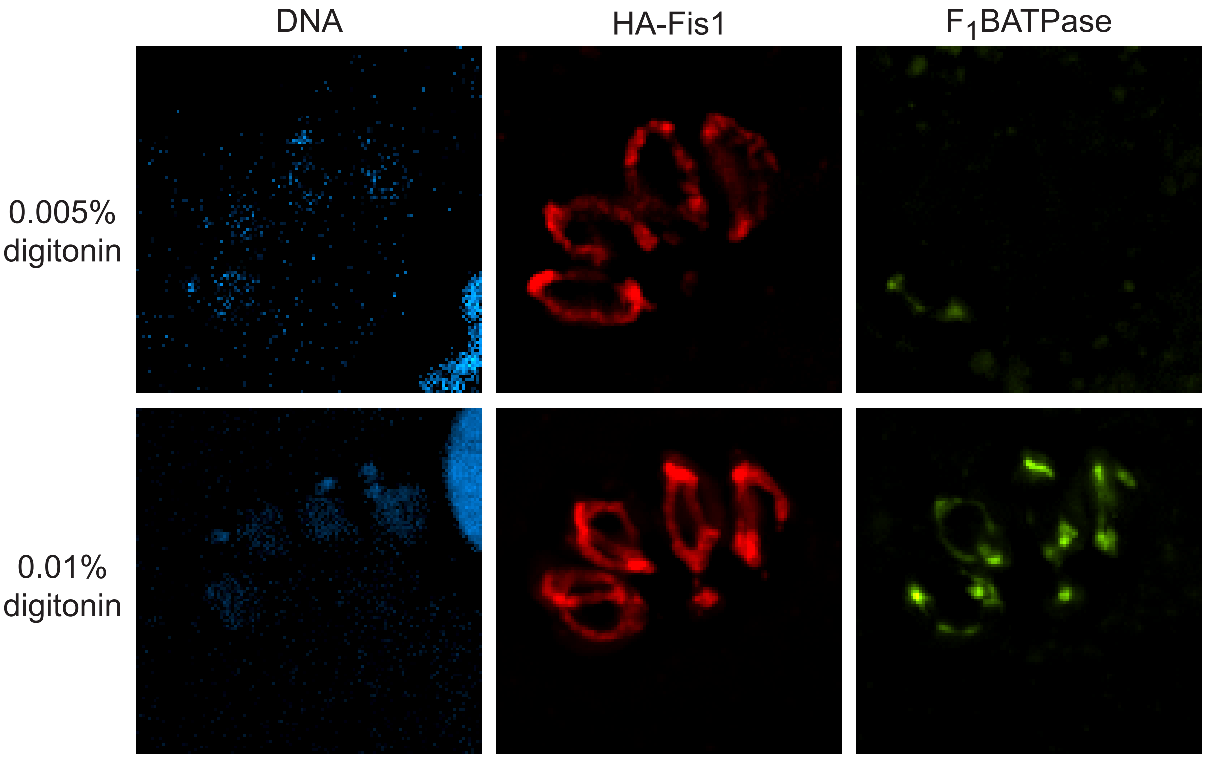

Supplemental figure S2. Intracellular parasites of the HA-Fis1 expressing strain were fixed and permeabilized with either 0.005% or 0.01% digitonin before staining for the IMM protein F1B ATPase (green) and HA (red). Fis1 can be detected when F1B ATPase remains inaccessible to the antibodies suggesting that it is associated with the OMM.
