# Supplementary material for: Identification of Fis1 Interactors in Toxoplasma gondii Reveals a Novel Protein Required for Peripheral Distribution of the Mitochondrion
Source: mBio. 2020 Feb 11;11(1):e02732-19. doi: 10.1128/mBio.02732-19 (PMC7018656; doi:10.1128/mBio.02732-19)
Supplement: FIG S3 [file mBio.02732-19-sf003.pdf]

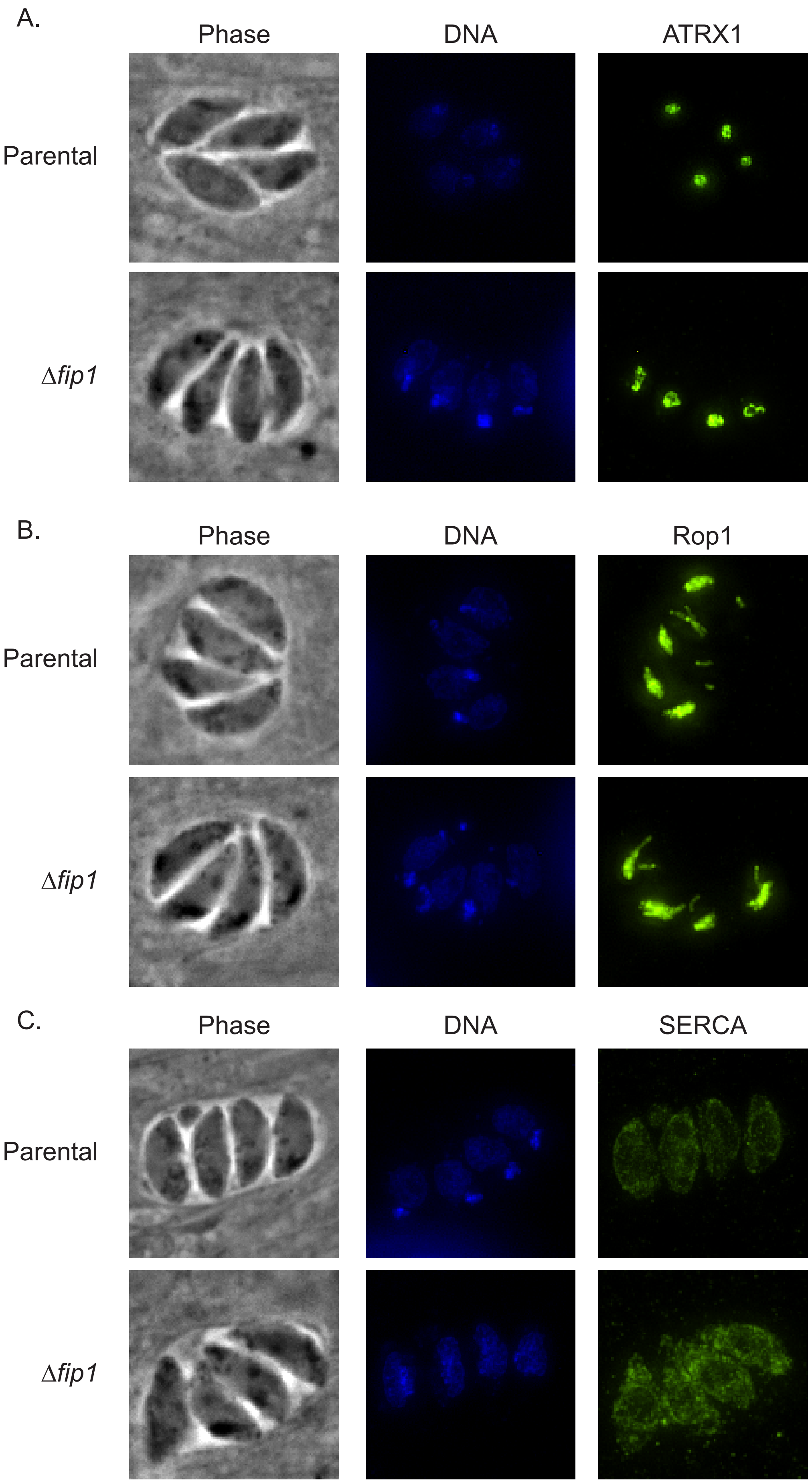

Supplemental figure S3. Intracellular parasites of the parental and the Δ265180 strains were stained with DAPI to visualized DNA and with antibodies against A. ATRX1 to visualize the apicoplast, B. Rop1 to visualize the rhoptries, and C. SERCA to visualize the endoplasmic reticulum.
