# Supplementary material for: Identification of Fis1 Interactors in Toxoplasma gondii Reveals a Novel Protein Required for Peripheral Distribution of the Mitochondrion
Source: mBio. 2020 Feb 11;11(1):e02732-19. doi: 10.1128/mBio.02732-19 (PMC7018656; doi:10.1128/mBio.02732-19)
Supplement: TABLE S2 [file mBio.02732-19-st002.pdf]

Use	Primer #	Name	Primer Sequence
Split N-terminal HA tag	1	RC #1	GTTTTTTGACGAGTATGCATATGTATCCTTACGATGTTCC
Ectopically express HA-Fis1	2	RC #2	CGATGTTCCAGATTATGCCGAAGACTCCAACTTCAG
	3	RC #3	GCACAACGGTGATTAATTAATTATTTGATAGCGTCCACAA
Ectopically express HA-Fis1ΔTM	4	RC #4	CGATGTTCCAGATTATGCCGAAGACTCCAACTTCAG
	5	RC #5	GCACAACGGTGATTAATTAATTAAAGCACGGAGCCAATGAG
Create Fis1 sgRNA for CRISPR	6	RC #6	ACTGGAGTCGGTTTTAGAGCTAGAAATAGCAAG
	7	RC #7	TTGAGTATGCAACTTGACATCCCCATTTAC
Endogenously tag TGGT1_265180 C-terminus with myc or HA	8	TgME49_265180 Tag.FOR	TTCCAATCCAATTTAATTAATGAGTACGCAGTGAGCCTTCG
	9	TgME49_265180 Tag.REV	CCACTTCCAATTTTAATTAACCAAAATCGAACCTTTCCGATGT
Ectopically express 265180-HA* and 265180ΔSID-HA under native promoter	10	FIP1 EE.HA.DHFR.FOR	TTCCAATCCAATTTAATTAATTATCTCCTTTGCGGACGCTAGG
	11	FIP1 no SID.REV	CCACTTCCAATTTTAATTAACTGCTTCGCGTCACTGC
Knockout of TGGT1_265180 coding sequence	12	Fis1.KO.5UTR.FOR	CGGTATCGATAAGCTTTTTTCGTTGAATCATATCCGCTTTGTCT
	13	Fis1.KO.5UTR.REV	CGTGCTGATCAAGCTTTCTCGTACAGTGCTCACAAAAAACGC
	14	Fis1.KO.3UTR.FOR	AGTTCTAGAGCGGCCGCATGGCGCCCCGGTTATCGGCTG
	15	Fis1.KO.3UTR.REV	ACCGCGGTGGCGGCCGCAGCGGTATACTAGAGGTCAGCGTACT
Confirmation of TGGT1_265180 knockout	16	Fis1 KO P1.FOR	TCAAGCAGACGGAGAGGC
	17	Fis1 KO P1.REV	CGACGGACTGTCCATACGT
	18	Fis1 KO P2-3.FOR	CAACTCACTGACCGCGGT
	19	Fis1 KO P3.REV	CAGAAGGGCTGTTGCGAG
Targeting to Ku80 site for complementation and ectopic expression	20	IMC2A-CmR [Ku80].F	GTCCCCGGTTCGCCTCAGCACACACACACATGACGTACATCGAAGCTGGGTACCCTGTACTTCC
	21	IMC2A-CmR [Ku80].R	GTAATGTCGGAATAGTTCCCATCAGAAACAATGGAGCTATCCGCGTCCCATTCGCCATTCAG
Mutating sgUPRT to sgKu80 for CRISPR/Cas9	22	sgKU80.F1	CTCATATTCCGTTTTAGAGCTAGAAATAGC
	23	sgKU80.R1	AAAGGTGTACAACTTGACATCCCCATTTAC

Table S2. Primers used in this study. All sequences are 5’ to 3’. *Used TgME49_265180 Tag.REV as the reverse primer for ectopic expression of 265180-HA
