# Supplementary material for: Identification of Fis1 Interactors in Toxoplasma gondii Reveals a Novel Protein Required for Peripheral Distribution of the Mitochondrion
Source: mBio. 2020 Feb 11;11(1):e02732-19. doi: 10.1128/mBio.02732-19 (PMC7018656; doi:10.1128/mBio.02732-19)
Supplement: DATA SET S2 [file mBio.02732-19-sd002.pdf]

For figure 6D

Probed with anti myc antibody

WT FISΔTM

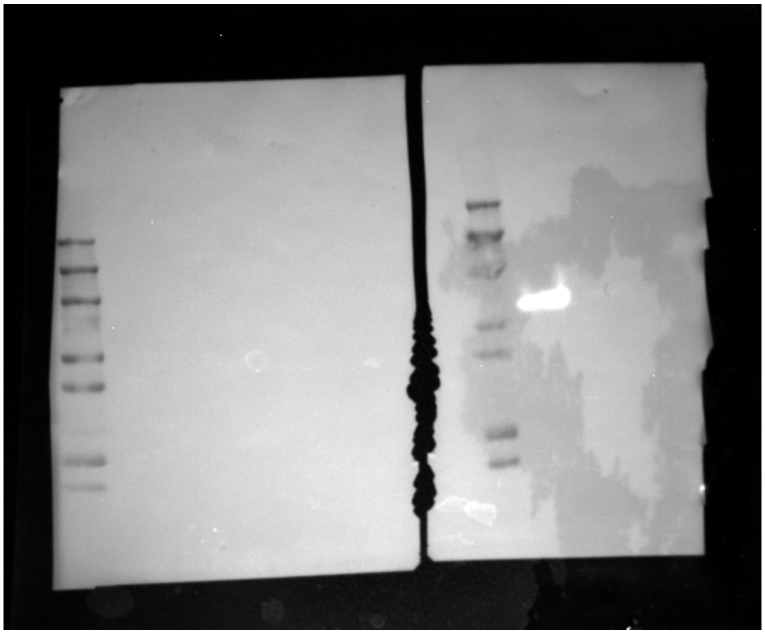

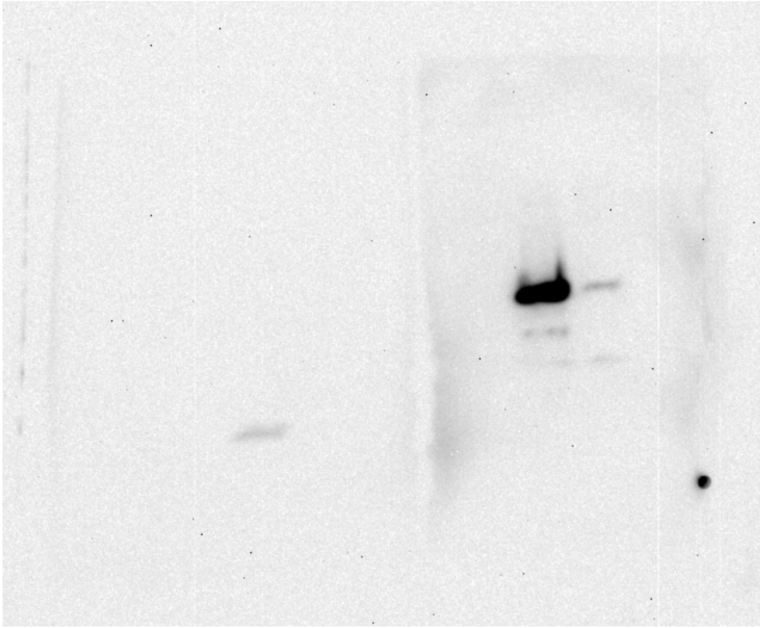

Probed with anti SAG1 antibody

WT FISΔTM

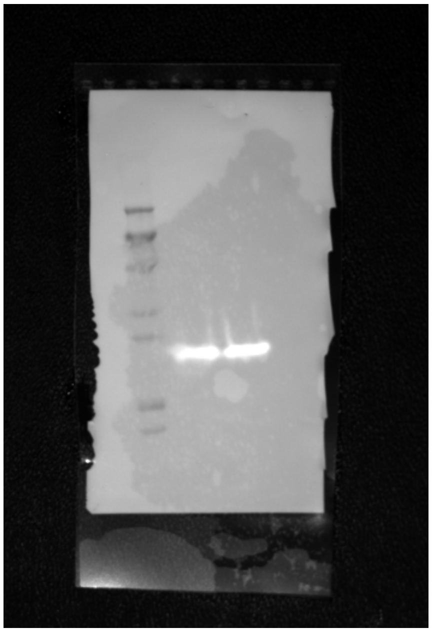

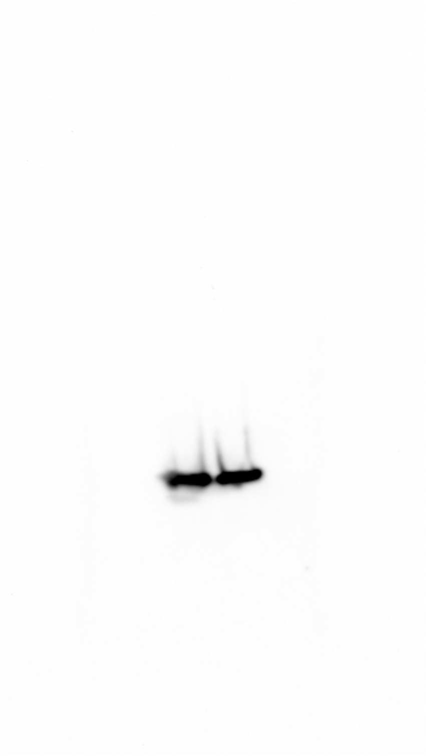

For figure 7D

Probed with anti HA antibody

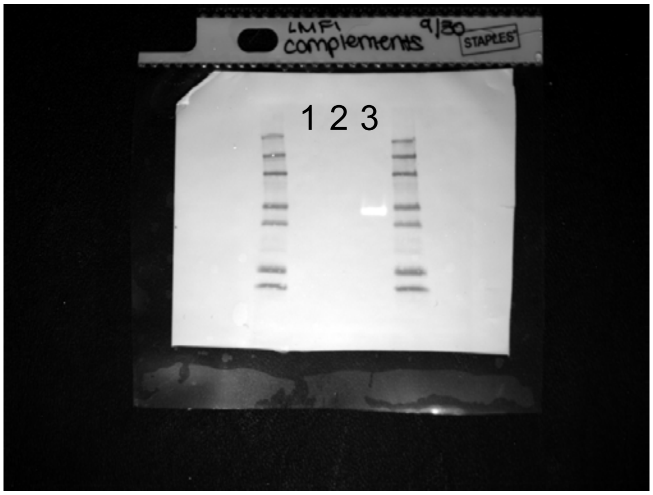


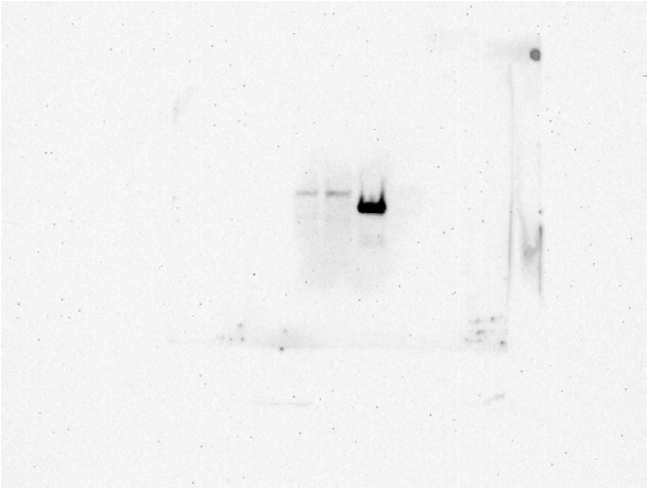

Probed with anti SAG1 antibody

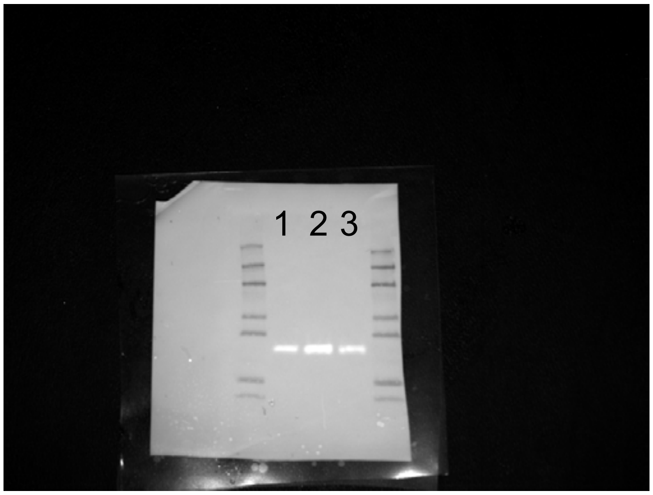


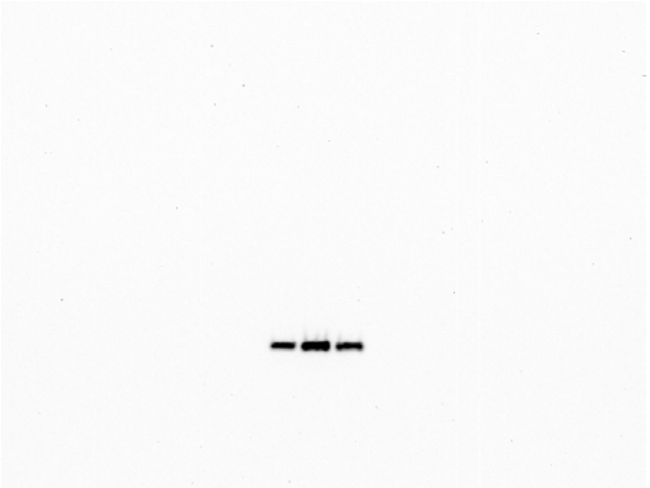

1: FIP-HA
2: ∆fip1+FIP1-HA
3: ∆fip1+FIP1∆SID-HA
